# The Role of the Complement System in Chronic Inflammatory Demyelinating Polyneuropathy: Implications for Complement-Targeted Therapies

**DOI:** 10.1007/s13311-022-01221-y

**Published:** 2022-04-04

**Authors:** Luis A. Querol, Hans-Peter Hartung, Richard A. Lewis, Pieter A. van Doorn, Timothy R. Hammond, Nazem Atassi, Miguel Alonso-Alonso, Marinos C. Dalakas

**Affiliations:** 1 Neuromuscular Diseases Unit, Department of Neurology, Hospital de La Santa Creu I Sant Pau, Barcelona, Spain; 2 Department of Neurology, Heinrich Heine University, Düsseldorf, Germany; 3 Brain and Mind Center, University of Sydney, Sydney, Australia; 4 Department of Neurology, Medical University of Vienna, Vienna, Austria; 5 Department of Neurology, Palacky University Olomouc, Olomouc, Czech Republic; 6 Cedars Sinai Medical Center, Los Angeles, CA USA; 7 Erasmus MC, University Medical Center, Rotterdam, The Netherlands; 8 Sanofi, Neurology Clinical Development, Cambridge, MA USA; 9 Department of Neurology, Thomas Jefferson University Hospital, Philadelphia, PA USA; 10 Neuroimmunology National and Kapodistrian University of Athens Medical School, Athens, Greece

**Keywords:** CIDP, Peripheral neuropathy, Demyelination, Pathogenesis, Complement system, Complement inhibition

## Abstract

Chronic inflammatory demyelinating polyneuropathy (CIDP) is the most common, heterogeneous, immune-mediated neuropathy, characterized by predominant demyelination of motor and sensory nerves. CIDP follows a relapsing–remitting or a progressive course and causes substantial disability. The pathogenesis of CIDP involves a complex interplay of multiple aberrant immune responses, creating a pro-inflammatory environment, subsequently inflicting damage on the myelin sheath. Though the exact triggers are unclear, diverse immune mechanisms encompassing cellular and humoral pathways are implicated. The complement system appears to play a role in promoting macrophage-mediated demyelination. Complement deposition in sural nerve biopsies, as well as signs of increased complement activation in serum and CSF of patients with CIDP, suggest complement involvement in CIDP pathogenesis. Here, we present a comprehensive overview of the preclinical and clinical evidence supporting the potential role of the complement system in CIDP. This understanding furnishes a strong rationale for targeting the complement system to develop new therapies that could serve the unmet needs of patients affected by CIDP, particularly in those refractory to standard therapies.

**Supplementary Information:**

The online version contains supplementary material available at 10.1007/s13311-022-01221-y.

## Introduction

Chronic inflammatory demyelinating polyneuropathy (CIDP; sometimes referred to as chronic inflammatory demyelinating polyradiculoneuropathy) is the most common and heterogeneous, immune-mediated peripheral neuropathy, typically characterized by predominant demyelination of motor and sensory nerves. CIDP manifests typically as an insidious onset of weakness, hypo- or areflexia, numbness, paresthesia, and sensory ataxia with a progressive or relapsing–remitting pattern [1–3]. The “typical” CIDP phenotype, characterized by symmetric and predominantly motor manifestations, is observed in about 50% patients [4]. “Atypical” CIDP, currently redefined as “CIDP variants,” comprises several well-characterized entities (multifocal, focal, distal, motor, or sensory CIDP) [3]. CIDP is closely related to Guillain-Barré Syndrome (GBS), another immune-mediated peripheral neuropathy that has an acute onset [5]. The estimated prevalence of CIDP varies between 0.8 and 10.3 cases per 100,000 people worldwide [6].

The current standard treatments for CIDP include intravenous immunoglobulins (IVIg), corticosteroids, and plasma exchange [4]. However, the response to these treatments remains partial/transient or requires long-term therapy [5]. CIDP is associated with substantial disability and loss of productivity [6]. An epidemiological study, conducted in the UK based on 2008 data, found that 32% of patients with CIDP were unable to walk independently [7]. The significant physical and psychological effects of CIDP markedly impact the patients’ quality of life [6]. According to a US nationwide survey in 475 patients with CIDP, 47% had to stop working due to the disease, and 20% had missed time from work/school due to symptoms [8]. Furthermore, studies have reported premature retirement in 14–28% of patients with CIDP [6].

A diverse and complex interplay of different immunopathological mechanisms, involving cellular, humoral and complement pathways, culminates in a highly variable pattern of peripheral nerve damage in different clinical variants of CIDP. Humoral factors and macrophage-mediated demyelination appear to play a crucial role in CIDP [9]; however, the triggers for these aberrant immune responses are not clearly understood [1, 9]. In some patients, macrophage infiltration in the myelinated fibers occurs around the nodes of Ranvier, while in other patients, the internodal region appears to be involved [2]. Deposition of antibody or complement at peripheral nerve components that distinguish the nodal regions may act as a trigger for macrophage-induced demyelination in certain subsets of patients [9].

In particular, the complement system appears to play an important role in CIDP pathogenesis. Studies have shown deposition of complement components, such as C3d and C9 neoantigen, as well as complement-fixing IgG and IgM on the myelin sheath in patients with CIDP [10]. Increased serum and cerebrospinal fluid (CSF) levels of C5a and the soluble terminal complement complex C5b-C9 have also been documented in patients with CIDP [11].

Most of the review articles discussing CIDP pathophysiology are focused on macrophage infiltration and demyelination and examined the role of autoantibodies directed to peripheral nerve components. However, there are no reviews centered on the role of the complement system in the pathogenesis of CIDP. Considering the current emergence and success of complement-targeted therapies in other autoimmune disorders, it is important to critically assess the role of the complement system and the suitability of complement-targeted therapeutics in CIDP patients.

## Pathophysiology of CIDP

**Fig. 1:**
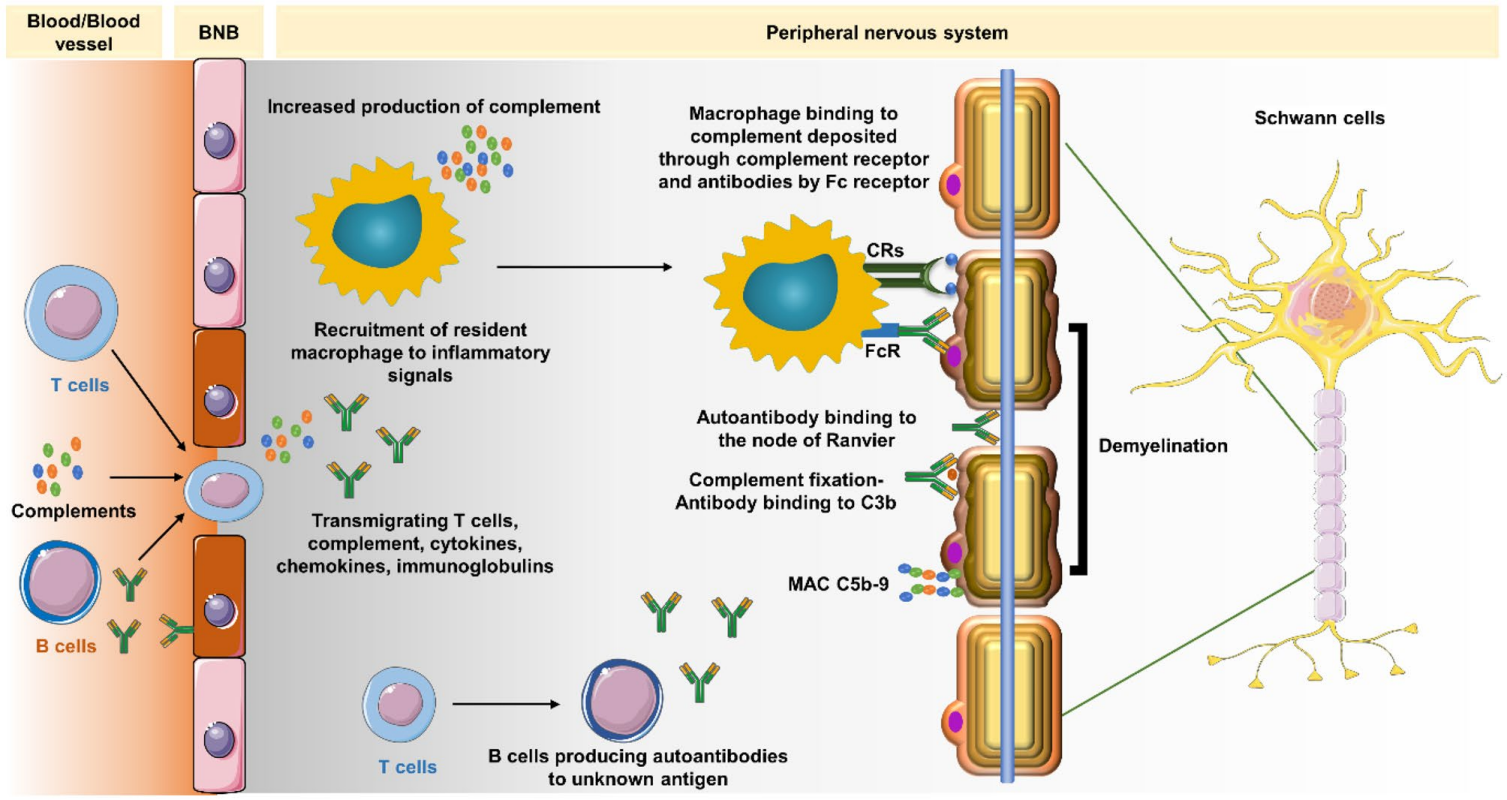
Role of complement in CIDP pathogenesis

Cellular and humoral components, including the complement system, are variably involved in peripheral nerve damage in CIDP (Fig. 1). However, the trigger(s) for the autoimmune response and the target antigen(s) involved remain elusive in most patients [12]. Histopathological changes in CIDP include breakdown of the blood–nerve barrier (BNB), segmental demyelination near the nodes of Ranvier, interstitial edema, and endoneurial inflammatory cell infiltration, including lymphocytes and macrophages and varying degrees of admixed axonal damage [1].

### Cellular Mechanisms

The involvement of cell-mediated immune mechanisms in CIDP is substantiated by evidence of T cell activation, the passage of activated T cells through the BNB, and the expression of pro-inflammatory cytokines [1]. Conventionally, macrophage and T-cell infiltration in the peripheral nerves and nerve roots and the subsequent segmental demyelination are believed to be the key cellular changes involved in CIDP pathogenesis [13].

Increased permeability of the BNB is the primary precursor for nerve inflammation and damage. While the exact mechanisms remain unknown, adhesion of activated T cells and upregulation of activation biomarkers and proinflammatory cytokines facilitate the enhanced permeability of the BNB, thus allowing transfer of macrophages and soluble factors, such as antibodies and complement including the C5b-9 membrane attack complex (MAC), to cross the BNB and exert nerve damage [12]. Furthermore, the severity and patterns of BNB damage correlate with CIDP phenotypes. Patients with typical clinical presentation of CIDP seem to have more severe BNB damage, compared with the other phenotypes, e.g., patients with typical CIDP have a prominent reduction in serum claudin-5 levels, a known paracellular permeability regulator [14]. In addition, there is a reduction in transendothelial electrical resistance (TEER) in peripheral nerve microvascular endothelial cells. These findings correlate with clinical and electrophysiological severity [14].

Macrophages are involved throughout the pathogenic process in autoimmune neuropathies and play an instrumental role in CIDP pathophysiology [15]. They are the dominant infiltrating cell population and cluster around endoneurial vessels [16]. Complement deposition is one of the facets of macrophage-mediated damage to neural tissue [17]. The presence of an array of complement receptors (CRs), including CR1, CR4, C3a anaphylatoxin receptor (C3aR), C5aR1, and C5aR2 [17] on the macrophages, may help facilitate myelin sheath damage and phagocytosis by these cells. Incidentally, the C3 receptor is also expressed by other immune cells, e.g., natural killer (NK) cells, monocytes, granulocytes [18], and neutrophils. Overall, this interconnection of different elements of the immune system further cements the role of the complement system in CIDP pathogenesis [19].

Sural nerve biopsies from CIDP patients also show signs of T cell infiltration [20]. Increased levels of T helper 17 (T_H_17) cells and expression of retinoic-acid-receptor-related orphan nuclear receptor gamma (RORγt) have been reported in the peripheral blood and CSF of patients with active CIDP, compared to those with remitting disease [21]. Additionally, patients with typical CIDP exhibit infiltrating CD8^+^ T cells with monoclonal and oligoclonal restrictions in sural nerve biopsies and peripheral blood [22, 23], indicating an antigen-driven response and perturbations of the T cell repertoire. Importantly, the myelinating Schwann cells in sural nerve biopsies from CIDP patients, but not the control nerves, express the co-stimulatory molecule BB-1 and possess the phenotypic markers of antigen-presenting cells [24]. Because their ligands CTLA-4 and CD28 are also overexpressed on the endoneurial T cells in the proximity to BB1-positive Schwann cells, the Schwann cells in CIDP patients have the capacity to function as professional antigen-presenting cells, possibly presenting endogenous or phagocytosed myelin antigens to T cells [24, 25].

The presence of cytokines may have implications in damaging the BNB and setting up a chemo-attractive environment for immune cell recruitment and subsequent neural insult [26]. Higher circulating levels of chemokines [27] and cytokines [28, 29] were reported in CIDP patients, which may increase BNB permeability and facilitate lymphoid cell infiltration. Elevated local expression of cytokines was also noted. Tumor necrosis factor-alpha (TNF-α), interferon-gamma (IFN-γ), and interleukin-2 (IL-2) were also observed within macrophages and T cells in sural nerve biopsies from CIDP patients.

Although direct evidence for a myelin antigen-specific auto-reactive T-cell population is currently lacking [30], a combined role of T cells and macrophages is very likely to be crucial in CIDP pathogenesis. Activated macrophages damaging the myelin sheath may lead to the release of other target molecules with subsequent broadening of autoantigenic epitopes, a process termed epitope spreading that creates an inflammatory cascade enhancing further the inflammatory milieus [30]. An interrelated and complex involvement of various immune components, including macrophages, T cells, activated antigen-presenting cells, cytokines, and the complement system, are proposed to be collectively involved in CIDP pathogenesis [31].

The involvement of T cells in CIDP pathogenesis is well established; however, the use of T cell-based therapies/inhibitors has not, to date, been shown to be efficacious in the management of CIDP. Fingolimod, a first in class sphingosine 1-phosphate receptor modulator that reduces the circulating naïve T- and B-cell population, has shown efficacy in multiple sclerosis. However, in the FORCIDP study, fingolimod did not demonstrate clinical benefit in CIDP, and the study was terminated prematurely for futility [32]. There are multiple reasons, including patient selection and study design, that may have affected the results, and more evidence will be required to rule out utility of T cell-based therapies in CIDP management. In addition, controlled trials with methotrexate, azathioprine, and interferons have also been unsuccessful in CIDP [33–35]. Anti B-cell therapies and therapies that target the neonatal Fc receptor (FcRn) can offer promising treatment options in CIDP, but further discussion around them is beyond the scope of this paper.

### Humoral Mechanisms

While myelin phagocytosis by macrophages is instrumental in CIDP pathogenesis, the triggers underlying this process are not clearly understood. It is postulated that deposition of autoantibodies specific to peripheral nerve antigens may be involved, via recognition of the immunoglobulin fragment crystallizable (Fc) portion or activation of the complement system [13].

The response to plasma exchange therapy indicates the important role of circulating factors in CIDP pathogenesis [12]; however, no specific target antigens have been identified in most patients [20, 31, 36]. Several studies attempted to elucidate the role of major myelin proteins, which are known to evoke experimental autoimmune neuritis (EAN), as the target antigens for autoantibodies in CIDP, but the results were not definitive [12]. Sera from only a minor proportion of CIDP patients demonstrated an antibody response to myelin protein zero (P0), myelin protein 2 (P2), and peripheral myelin protein (PMP)-22, although IgG antibodies were significantly higher in patients with CIDP than in controls [37]. Antibodies to acidic glycolipids were also more frequently seen in CIDP patients [5, 38, 39]. However, in the majority of these patients, no pathogenic antibodies are identified.

Antibodies targeting nodes of Ranvier structures have recently been identified [40–42]. Antibodies against the paranodal protein contactin-1 (CNTN1) were identified in a subset of CIDP patients [43]. Antibodies to Caspr1 were also detected in CIDP patients but not in healthy controls and in patients with other neuropathies [44]. Further, antibodies to Caspr1/CNTN1 complex were also recognized in a subset of CIDP patients with similar serological and clinical manifestations [45]. Antibodies against neurofascin-155 (NF-155), a glial counterpart of CNTN1, were demonstrated in another subset of CIDP patients [46]. It is noteworthy that almost all the antibodies to these paranodal antigens were IgG4, an IgG subclass that is non-complement fixing [47]. This may partially explain the lack of treatment response to IVIg treatment in this small subset of CIDP patients who carry these antibodies [46, 47]. Nonetheless, some variants of these neuropathies harbor IgG1-3 subclass of antibodies with ability to fix complement and could potentially be candidates to complement inhibition [48–51]. A systematic screening of antibodies revealed a diverse antibody repertoire in CIDP patients, including those against NF-155 (4.6%), CNTN1 (6.2%), Caspr1/CNTN1 complex (1.5%), P2 (1.6%), and anti-ganglioside antibodies (18.6%). In the great majority of CIDP, patients target antigens of an antibody response remain still elusive [5, 39, 52, 53]. Such a diverse antibody profile further strengthens the heterogeneity in CIDP pathogenic mechanisms [20, 31, 40].

Activation of the complement system by autoantibodies may contribute to various mechanisms inflicting damage to the myelin sheath. One is to facilitate macrophage-mediated myelin phagocytosis, [13], an observation that has been described in a patient with antibodies against the sialosylneolactotetraosylceramide (LM1) glycolipid [54].

The efficacy of plasma exchange in CIDP further substantiates the role of circulating factors, and potentially that of the complement system, in the disease pathogenesis [12]. A study using sera obtained from patients with CIDP who responded to plasma exchange showed binding of IgG and C3d in 4 of 12 patients [55]. However, complement involvement in certain subgroups of CIDP patients, including those with non-complement fixing antibodies, is not yet certain [31, 56].

## Role of the Complement Pathway in CIDP

The complement system is an integral part of innate immune defense and plays an important physiological role in tissue remodeling [56]. The products of complement activation act as a link between the innate and the adaptive immune systems, by acting directly on receptors expressed on T cells, B cells, and macrophages or by modulating dendritic cell functions.

Complement activation occurs through three different pathways: classical (C1q), lectin (mannose-binding lectins or ficolin), and alternative (C3 autoactivation or properdin), which converge at C3, the most abundant complement protein found in the blood [56]. Subsequently, the effector proteins C3a, C3b, and C5a and the membrane attack complex (MAC, C5b-9) are generated for target cell lysis [56, 57].

Complement is a complex system, and disruption of its delicate regulation triggers several autoimmune neurological disorders [56]. The role of the complement system in various neuropathies and neurodegenerative diseases, including Guillain–Barré syndrome (GBS), has been well documented. GBS is an inflammatory demyelinating polyneuropathy which, due to significantly overlap in electrophysiological, histological, and clinical features, is considered to be an acute clinical counterpart of CIDP. However, CIDP and GBS differ in triggers, time course, and response to corticosteroid treatment [20]. In acute inflammatory demyelinating neuropathy (AIDP), the predominant phenotype of GBS, an autopsy study showed complement activation and MAC deposition on Schwann cell membranes [58]. In the animal model of experimental autoimmune neuritis, deposition of MAC preceded demyelination [59]. Moreover, C3 depletion has been shown to reduce demyelination and inflammation in experimental allergic neuritis [60, 61]. In acute motor axonal neuropathy (AMAN), another phenotype of GBS, *Campylobacter jejuni* infection-induced autoantibodies have been found to activate complement and MAC deposition at the nodes of Ranvier [62].

These data suggest that complement-mediated processes in peripheral neuropathies are important mediators in their pathobiology. Hence, the therapeutic approach of targeting the complement system seems to be viable [31, 63]. In addition, success with complement inhibition with eculizumab in Myasthenia gravis, an autoimmune neuromuscular condition, [64, 65] encourages exploring this pathway as a potential treatment target in other autoimmune conditions. In the following sections, we review the preclinical and clinical evidence regarding the role of complement in CIDP.

## Preclinical Evidence of Complement Involvement in CIDP

Complement plays a role in various autoimmune neuropathies, but much of this data comes from studies on various animal models of peripheral nerve inflammation [15]. The EAN rat model is a widely accepted model for GBS, but some of the inflammatory mechanisms modeled in EAN are also shared with CIDP [55]. This model has been used extensively since it replicates pathological hallmarks such as macrophage-mediated stripping and phagocytosis of compact myelin lamellae, which are observed in CIDP [66]. In this model, cobra venom factor (CVF), which is known to deplete C3, was found to reduce EAN severity, possibly by blocking antigen–antibody complex-induced activation of complement [60]. Depletion of complement with CVF reduced clinical scores, demyelination, and inflammation [61, 67]. The role of complement in autoimmune neuropathies was further validated by administration of soluble CR1, a natural complement inhibitor that binds and promotes degradation of C3b and C4b, resulting in suppression of clinical signs of disease in rats with EAN [68]. Additional evidence strengthening complement activation in CIDP includes blockade of nerve conduction with administration of patient-derived pathogenic IgG with C3 reactivity [55] and demyelination and conduction blockade due to intraneural administration of patient-derived anti-P0 IgG antibodies [69].

Evidence is also available on the role of complement in other animal studies of peripheral neuropathies. In a mice model of AMAN, inhibition of C1q (with an anti-C1q antibody) resulted in attenuation of complement cascade activation and complement deposition, as demonstrated by absence of C3c staining or C5b-9 components at the nerve terminals. There was also evidence of reduced immune cell recruitment and reduced axonal injury [70].

A novel “Human-in-a-chip” in vitro model, using a microelectrode array with directed axonal outgrowth over the electrodes, was recently developed. A human CIDP model was created by treatment of Schwann cell and motoneuron cultures with CIDP patient-derived serum, which triggered classical complement pathway activation and, subsequently, the deposition of C3b and C5b-9. Furthermore, treatment with a test monoclonal antibody inhibitor of C1s, TNT005, resulted in abrogation of complement deposition and rescued the electrophysiological deficits [71].

## Clinical Evidence of Complement Involvement in CIDP

Complement involvement in CIDP pathogenesis is also substantiated by several lines of histopathological, serological and clinical evidence.

### Genetic Data

The study of CIDP-related genetic factors remains limited due to the rarity of the disease and heterogeneity in the CIDP phenotypes [72]. Cases of CIDP variants have been described in infants that do not meet the diagnostic criteria but nevertheless respond to immunomodulatory therapy. A case series described such CIDP-like illness in five infants with inherited chronic hemolysis. Although these patients did not have typical CIDP, the relapsing nature of their disease, demyelination, and improvement in upper limb muscle strength with immunomodulatory treatment mimicked CIDP. Treatments with IVIg, corticosteroids, rituximab, and cyclosporine, while failing to prevent relapses, shortened their duration and lessened their severity. Homozygosity mapping, followed by exome sequencing, revealed a homozygous missense mutation, p.Cys89Tyr, in CD59 (protectin) in all of these patients. Further analysis in four of five patients (one deceased) revealed that there was a lack of CD59 expression on red blood cell (RBC) membranes, monocytes, and lymphocytes. CD59 normally protects host cells from complement MAC-mediated injury by binding to C8 and C9 and preventing pore formation in the nerve cell membrane. C59 deficiency has been associated with demyelination via MAC activation, and MAC is known to play a vital role in demyelination in early-onset CIDP [73]. Four pediatric patients with recurrent demyelinating neuropathy featuring conduction block and p.Cys89Tyr missense mutation in the CD59 gene, unresponsive to IVIg and steroids, had a dramatic improvement of the neuropathy after eculizumab, a monoclonal antibody preventing activation of C5 and subsequently assembly of the MAC [74]. Overall, the CD59 loss of function mutations in case series of patients with CIDP-like phenotype, along with the response to complement inhibition, suggests a direct link between compliment activation and acquired chronic peripheral nerve demyelination.

Based on these observations, a hypothesis that de novo CD59 mutations may be present in patients with sporadic CIDP was tested. A study performed direct sequencing of all coding exons of CD59 in 35 adult patients with sporadic CIDP. However, the p.Cys89Tyr mutation was not identified in any of the patients. Only one variant, a heterozygous guanine to adenine substitution (c.18G > A), was noted but was not shown to have any deleterious effect [72]. Considering the small sample size, these findings cannot completely rule out CD59 mutation in some rare cases of CIDP, and larger studies are required to arrive at a definitive conclusion.

### Complement Deposition in Nerves

**Fig. 2:**
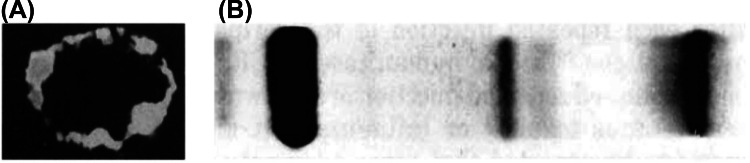
Complement deposition in sural nerve biopsy from CIDP patient (**A**) and agarose gel electrophoresis patterns of CSF in a patient with chronic relapsing biopsy (**B**). **A**: Reproduced from Dalakas MC and Engel WK [10]; **B**: Reproduced from Dalakas et al [76]

Evidence of complement involvement is supported by the demonstration of complement deposition in sural nerve biopsies from CIDP patients. In an early study, sural nerve biopsy specimens from seven patients with chronic relapsing demyelinating polyneuropathy showed deposition of IgM in intraneural blood vessels in all patients and deposition of C3 in six of the seven patients. Deposits of IgM, but not of C3, were also noted on the Schwann cell plasmalemma of even non-demyelinated nerve fibers in CIDP patients (Fig. 2A). However, no such deposition was noted in control patients. These complement-fixing immunoglobulin deposits in intact appearing nerve fibers may contribute to nerve injury by inducing vascular permeability changes, thereby increasing BNB permeability, and enhance the access of antibodies to nerve fibers [10]. In another study, sural nerve biopsies on specimens from 100 patients with neuropathy or motor neuron disease were conducted to analyze both C3d and C3c complement components. Among 4 of the 100 patients who had chronic relapsing polyneuropathy, C3d deposition was noted on the surface of some myelin sheaths in 2 patients [75]. The presence of C3d as a part of the immune complex on myelin sheaths suggests a role of the complement system in the development of neuropathy.

A recent case report of a patient with CIDP demonstrated presence of a circulating IgG antibody to LM1, but not to NF-155, CNTN-1, GM1, and GD1b. An immunohistochemical analysis revealed deposition of C9 neoepitope, a component of MAC, on the compact myelin sheath. Macrophage infiltration was evident with the presence of several CD68-positive cells in each fascicle. Furthermore, deposition of complement was noted at the internodes, which comprise the majority of the length of myelinated fibers. This case highlights the role of complement-dependent cytotoxicity in the pathogenesis of CIDP with anti-LM1 antibodies [54].

### Complement Pathway Activation in Serum/CSF

Apart from complement deposition on the myelin sheath, complement components have also been identified in serum and CSF samples from patients with CIDP. A study compared serum and CSF levels of C5a and terminal complement complex (TCC) in 21 newly diagnosed, treatment-naïve CIDP patients, with that of 29 control patients with noninflammatory neurological diseases. CIDP patients had significantly higher mean serum and CSF levels of C5a and TCC than control patients [11]. This is also supported by the finding of a stable IgG band in the CSF of CIDP patients suggesting of persistent antigenic stimulation **(**Fig. 2B) [76, 77]. Results also indicated a systemic rather than local CSF activation of the complement system in CIDP [11].

Complement components also appear to correlate with CIDP disease activity. In CIDP patients, systemic terminal complement activation was associated with residual disease severity after 4 weeks of IVIg treatment. In 80% of these patients, inflammatory neuropathy cause and treatment (INCAT) disability scores were reduced after IVIg treatment for 4 weeks. CIDP patients with higher disability at the time of diagnosis tended to have higher serum C5a and soluble TCC levels; these levels reduced and significantly correlated with the reduction in INCAT scores at the week 4 follow-up visit after initiation of IVIg treatment. Larger studies are warranted to examine terminal complement activation as a potential surrogate marker for clinical disease activity, progression, and response to immunomodulatory therapies in patients with major phenotypic variants of CIDP [11].

### Complement Involvement in GBS

As noted previously, both GBS and CIDP exhibit similar patterns of macrophage-mediated demyelination originating from similar, pathogenic mechanisms [10, 20, 31] like CIDP, the understanding of immunopathogenesis in GBS is derived from limited pathological data and acute EAN models [13]. Hence, evidence of complement involvement in GBS indirectly strengthens the role of the complement system in CIDP.

Complement involvement has been implicated among one of the diverse mechanisms of GBS pathogenesis and is validated by adequate clinical evidence. The presence of C3d was reported on the Schwann cell plasma membrane in myelinated fibers, but not in unmyelinated fibers, in autopsy studies of three GBS patients. Immunocytochemistry revealed that in many nerve fibers, there was a rim of C3d and C5b-9 along the outer surface of the Schwann cells. Further analysis of such C3d-positive fibers revealed mild vesicular changes in the outermost myelin lamellae [78]. Increased serum TCC levels in GBS patients have been reported to decline with clinical improvement, becoming undetectable a month after symptom onset. Membrane-bound TCC was also found deposited on the abaxonal Schwann cell surface in a GBS patient [79]. Immunohistochemistry of sural nerve biopsies from five AIDP (a form of GBS) patients demonstrated C3d or C9 neoepitope deposition in the endoneurium [80]. Similarly, elevated CSF levels of C3a and C5a have been reported in GBS patients [81]. These findings indicate complement cascade activation due to binding of specific antibodies to the abaxonal Schwann cell surface in GBS [78].

The accumulating evidence regarding complement involvement in many autoimmune neuropathies has generated great interest and encouraged clinical research focused on complement-targeted therapies in several neurological disorders [56]. Complement inhibition with eculizumab, a humanized monoclonal antibody to C5, is currently being investigated in GBS and has demonstrated some clinical benefit in phase 2 studies. In one phase 2 study, the primary efficacy outcome (proportion of patients who reach functional grade ≤ 2) was not achieved, but a clinical benefit was observed in secondary efficacy outcomes (proportion of patients who could run at week 24) [82]. The results of another phase 2 study were inconclusive considering the small sample size and severity of disease in enrolled patients [83]. A phase 3 study to evaluate the efficacy and safety of eculizumab in GBS (NCT04752566) is ongoing. Additional complement inhibitors that target different components of the pathway are in development [56] and will provide the opportunity to test the complement’s role in GBS and CIDP. Two classical pathway inhibitors are in clinical trials for GBS and CIDP. For example, a phase 2/3 study for ANX005, a C1q inhibitor, in GBS is currently ongoing (NCT04701164), and a phase 2 study for an inhibitor of active C1s is also underway in CIDP (NCT04658472).

## Discussion/Summary

CIDP is the most prevalent chronic autoimmune neuropathy and causes substantial disability [6, 20]. The pathogenesis of CIDP remains elusive, however, a complex interplay of multiple immunological pathways has been documented. The available literature on CIDP pathogenesis appears to be focused on macrophage-mediated demyelination and attempts to identify autoantibodies. However, a careful consideration of the observations on complement in various studies highlights an intriguing role for complement in CIDP pathogenesis **(**Fig. 1).

A comprehensive review of studies aimed at elucidating the pathogenic mechanisms underlying CIDP reveals evidence of activation of complement both systemically and in peripheral nerve. Data from the EAN model-based preclinical studies has demonstrated reduced severity [60], demyelination, and inflammation [61, 67] with complement depletion. Passive transfer of the disease with pathogenic IgG with complement C3 reactivity from CIDP patients into rats, resulting in nerve conduction impairment and demyelination [55], and suppression of disease following administration of sCR1 [68], indicates a pathogenetically relevant association of complement in CIDP patients. Furthermore, deposition of complement components on patients' nerves [10, 75], complement-fixing autoantibodies on the myelin sheath [54], and high systemic complement levels that correlated with disease severity [11] further underscore a role of complement in CIDP pathophysiology. Complement capture and inhibition are among the mechanisms of action of IVIg, the currently most effective and widely used therapy in CIDP [20, 31, 47]. Moreover, genetic mutation leading to deficiency of CD59, a complement regulator, resulting in CIDP-like illness in infants [73], provides further supporting evidence. Collectively, these data provide arguments to invoke complement as a key mediator of nerve dysfunction and damage in CIDP, at least in a subset of CIDP patients.

The standard of care (SOC) therapies in CIDP, though effective, have several limitations, such as variable treatment response, side effects with chronic use, need for venous access, dependency on plasma donations (IVIg) leading to potentially limited availability, and high patient burden. Various clinical phenotypes of CIDP appear to have similar but not identical, pathogenic causes, which may explain the variability in treatment response to the SOC therapies. There is, therefore, a substantial unmet need in CIDP treatment that requires the development of alternative therapies that reflect the various disease phenotypes and thereby offers treatments targeted to individual patients.

The lack of efficacy of fingolimod, a T-cell-based therapy [32], has encouraged consideration of the complement system and other potential immune mechanisms in CIDP [56]. With its emergence of a likely important role in CIDP, complement has now become an attractive therapeutic target.

Several complement inhibitors, each targeting a specific step in the complement pathway, are under development, and clinical studies are ongoing to explore their potential clinical benefits [56, 84]. Eculizumab, a humanized monoclonal antibody that inhibits C5, has shown clinical benefits in generalized myasthenia gravis [23] and is currently being investigated in GBS (NCT04752566). A safety and efficacy study for ANX005, a C1q inhibitor, in GBS (NCT04701164) is also underway. Furthermore, an active C1s inhibitor, SAR445088, is currently being investigated in CIDP (NCT04658472).

Pre-clinical research in CIDP remains limited due to lack of disease-specific animal models. Conducting clinical trials is cumbersome due to difficulties in patient recruitment, disease heterogeneity, and the lack of objective biomarkers to characterize the disease and evaluate response to therapies. Hence, further clinical research in CIDP can benefit from the use of novel approaches in study design to overcome these challenges and ensure solid conclusions when testing new treatment options in this field.

In summary, considering the drawback of the current SOC therapies, the preclinical and clinical evidence supporting the role of complement in CIDP pathogenesis, and the plausible success of early stage complement inhibitors in diseases other than CIDP, exploring the benefits of complement pathway inhibitors in CIDP is an attractive proposition.

## Supplementary Information

Below is the link to the electronic supplementary material.

Supplementary file1 (PDF 527 KB)

Supplementary file2 (PDF 2081 KB)

Supplementary file3 (PDF 517 KB)

Supplementary file4 (PDF 1225 KB)

Supplementary file5 (PDF 517 KB)

Supplementary file6 (PDF 535 KB)

Supplementary file7 (PDF 509 KB)

Supplementary file8 (PDF 534 KB)
