# Supplementary material for: The Role of the Complement System in Chronic Inflammatory Demyelinating Polyneuropathy: Implications for Complement-Targeted Therapies
Source: Neurotherapeutics. 2022 Apr 4;19(3):864–73. doi: 10.1007/s13311-022-01221-y (PMC9294101; doi:10.1007/s13311-022-01221-y)
Supplement: Supplementary file 2 — Supplementary file2 (PDF 2081 KB) [file 13311_2022_1221_MOESM2_ESM.pdf]

ICMJE INTERNATIONAL COMMITTEE of MEDICAL JOURNAL EDITORS

ICMJE Form for Disclosure of Potential Conflicts of Interest

Instructions

The purpose of this form is to provide readers of your manuscript with information about your other interests that could influence how they receive and understand your work. The form is designed to be completed electronically and stored electronically. It contains programming that allows appropriate data display. Each author should submit a separate form and is responsible for the accuracy and completeness of the submitted information. The form is in four parts.

1. Identifying information.

Enter your full name. If you are NOT the corresponding author please check the box "no" and a space to enter the name of the corresponding author in the space that appears. Provide the requested manuscript information. Double-check the manuscript number and enter it.

2. The work under consideration for publication.

This section asks for information about the work that you have submitted for publication. The time frame for this reporting is that of the work itself, from the initial conception and planning to the present. The requested information is about resources that you received, either directly or indirectly (via your institution), to enable you to complete the work. Checking "No" means that you did the work without receiving any financial support from any third party -- that is, the work was supported by funds from the same institution that pays your salary and that institution did not receive third-party funds with which to pay you. If you or your institution received funds from a third party to support the work, such as a government granting agency, charitable foundation or commercial sponsor, check "Yes". Then complete the appropriate boxes to indicate the type of support and whether the payment went to you, or to your institution, or both.

3. Relevant financial activities outside the submitted work.

This section asks about your financial relationships with entities in the bio-medical arena that could be perceived to influence,or that give the appearance of potentially influencing, what you wrote in the submitted work. You should disclose interactions with ANY entity that could be considered broadly relevant to the work. For example, if your article is about testing an epidermal growth factor receptor (EGFR) antagonist in lung cancer, you should report all associations with entities pursuing diagnostic or therapeutic strategies in cancer in general, not just in the area of EGFR or lung cancer.

Report all sources of revenue paid (or promised to be paid) directly to you or your institution on your behalf over the 36 months prior to submission of the work. This should include all monies from sources with relevance to the submitted work, not just monies from the entity that sponsored the research. Please note that your interactions with the work's sponsor that are outside the submitted work should also be listed here. If there is any question, it is usually better to disclose a relationship than not to do so.

For grants you have received for work outside the submitted work, you should disclose support ONLY from entities that could be perceived to be affected financially by the published work, such as drug companies, or foundations supported by entities that could be perceived to have a financial stake in the outcome. Public funding sources, such as government agencies, charitable foundations or academic institutions, need not be disclosed. For example, if a government agency sponsored a study in which you have been involved and drugs were provided by a pharmaceutical company, you need only list the pharmaceutical company.

4. Other relationships.

Use this section to report other relationships or activities that readers could perceive to have influenced, or that give the appearance of potentially influencing, what you wrote in the submitted work.

INTERNATIONAL COMMITTEE of MEDICAL JOURNAL EDITORS

ICMJE Form for Disclosure of Potential Conflicts of Interest

Section 1. Identifying Information

1. Given Name (First Name): Pieter
2. Surname (Last Name): van Doorn
3. Effective Date (07-August-2008): 19-January-2022

4. Are you the corresponding author? ☐ Yes ☑ No

Corresponding Author's Name: Marinos Dalakas

5. Manuscript Title: The Role of Complement System in Chronic Inflammatory Demyelinating Polyneuropathy

6. Manuscript Identifying Number (if you know it)

Section 2. The Work Under Consideration for Publication

Did you or your institution at any time receive payment or services from a third party for any aspect of the submitted work (including but not limited to grants, data monitoring board, study design, manuscript preparation, statistical analysis, etc...)?

Complete each row by checking "No" or providing the requested information. If you have more than one relationship click the "Add" button to add a row. Excess rows can be removed by clicking the "X" button.

The Work Under Consideration for Publication

Type	No	Money Paid to You	Money to Your Institution*	Name of Entity	Comments**	
1. Grant	☑	☐	☐			X ADD
2. Consulting fee or honorarium	☑	☐	☐			X ADD
3. Support for travel to meetings for the study or other purposes	☑	☐	☐			X ADD
4. Fees for participation in review activities such as data monitoring boards, statistical analysis, end point committees, and the like	☑	☐	☐			X ADD
5. Payment for writing or reviewing the manuscript	☑	☐	☐			X ADD
6. Provision of writing assistance, medicines, equipment, or administrative support	☑	☐	☐			X

ICMJE | INTERNATIONAL COMMITTEE of MEDICAL JOURNAL EDITORS

ICMJE Form for Disclosure of Potential Conflicts of Interest

The Work Under Consideration for Publication						
Type	No	Money Paid to You	Money to Your Institution*	Name of Entity	Comments**	
						ADD
7. Other	✓	☐	☐			×
						ADD

* This means money that your institution received for your efforts on this study.
** Use this section to provide any needed explanation.

Section 3. Relevant financial activities outside the submitted work.

Place a check in the appropriate boxes in the table to indicate whether you have financial relationships (regardless of amount of compensation) with entities as described in the instructions. Use one line for each entity; add as many lines as you need by clicking the "Add +" box. You should report relationships that were present during the 36 months prior to submission.

Complete each row by checking "No" or providing the requested information. If you have more than one relationship click the "Add" button to add a row. Excess rows can be removed by clicking the "X" button.

Relevant financial activities outside the submitted work						
Type of Relationship (in alphabetical order)	No	Money Paid to You	Money to Your Institution*	Entity	Comments	
1. Board membership	✓	☐	☐			×
						ADD
2. Consultancy	☐	☐	✓	Argenx, Sanofi, Octapharma, Annexon, Roche, Immunics	Advise/Steering committee clinical studies/RCT's	×
						ADD
3. Employment	✓	☐	☐			×
						ADD
4. Expert testimony	✓	☐	☐			×
						ADD
5. Grants/grants pending	☐	☐	✓	Prinses Beatrix Spierfonds	Clinical studies	×
5. Grants/grants pending	☐	☐	✓	Netherlands Organisation for Health Research and Development (ZonMW)	Research project	×

ICMJE INTERNATIONAL COMMITTEE *of* MEDICAL JOURNAL EDITORS

ICMJE Form for Disclosure of Potential Conflicts of Interest

Relevant financial activities outside the submitted work

Type of Relationship (in alphabetical order)	No	Money Paid to You	Money to Your Institution*	Entity	Comments	
5. Grants/grants pending	☐	☐	☑	Takeda	Clinical studies/RCT	×
5. Grants/grants pending	☐	☐	☑	Sanquin Blood Supply	RCT	× ADD
6. Payment for lectures including service on speakers bureaus	☑	☐	☐			× ADD
7. Payment for manuscript preparation	☑	☐	☐			× ADD
8. Patents (planned, pending or issued)	☑	☐	☐			× ADD
9. Royalties	☑	☐	☐			× ADD
10. Payment for development of educational presentations	☑	☐	☐			× ADD
11. Stock/stock options	☑	☐	☐			× ADD
12. Travel/accommodations/ meeting expenses unrelated to activities listed**	☑	☐	☐			× ADD
13. Other (err on the side of full disclosure)	☑	☐	☐			× ADD

* This means money that your institution received for your efforts.

** For example, if you report a consultancy above there is no need to report travel related to that consultancy on this line.

INTERNATIONAL COMMITTEE of MEDICAL JOURNAL EDITORS

ICMJE Form for Disclosure of Potential Conflicts of Interest

Section 4. Other relationships

Are there other relationships or activities that readers could perceive to have influenced, or that give the appearance of potentially influencing, what you wrote in the submitted work?

- [x] No other relationships/conditions/circumstances that present a potential conflict of interest
- [ ] Yes, the following relationships/conditions/circumstances are present (explain below):

At the time of manuscript acceptance, journals will ask authors to confirm and, if necessary, update their disclosure statements. On occasion, journals may ask authors to disclose further information about reported relationships.

Hide All Table Rows Checked 'No'   SAVE

Evaluation and Feedback

Please visit http://www.icmje.org/cgi-bin/feedback to provide feedback on your experience with completing this form.
